# A Chinese patient with peritoneal dialysis-related peritonitis caused by *Gordonia terrae*: a case report

**DOI:** 10.1186/s12879-017-2283-2

**Published:** 2017-02-28

**Authors:** Chenrui Hou, Yun Yang, Ziyang Li

**Affiliations:** Department of Clinical Laboratory, The Shanxi Dayi Hospital, 99 Longcheng Road, Taiyuan, 030032 Shanxi China

**Keywords:** Peritonitis, Peritoneal dialysis, *Gordonia terrae*, Case report

## Abstract

**Background:**

*Gordonia terrae* is a rare cause of clinical infections, with only 23 reported cases. We report the first case of peritoneal dialysis-related peritonitis caused by *Gordonia terrae* in mainland China.

**Case presentation:**

A 52-year-old man developed peritoneal dialysis-related peritonitis and received preliminary antibiotic treatment. After claiming that his symptoms had been resolved, the patient insisted on being discharged (despite our recommendations) and did not receive continued treatment after leaving the hospital. A telephone follow-up with the patient’s relatives revealed that the patient died 3 months later. Routine testing did not identify the bacterial strain responsible for the infection, although matrix-assisted laser desorption/ionization time-of-flight mass spectrometry identified the strain as *Gordonia rubropertincta*. However, a 16S rRNA sequence analysis using an isolate from the peritoneal fluid culture revealed that the responsible strain was actually *Gordonia terrae*. Similar to this case, all previously reported cases have involved a delayed diagnosis and initial treatment failure, and the definitive diagnosis required a 16S rRNA sequence analysis. Changes from an inappropriate antibiotic therapy to an appropriate one have relied on microbiological testing and were performed 7–32 days after the initial treatment.

**Conclusions:**

The findings from our case and the previously reported cases indicate that peritoneal dialysis-related peritonitis caused by *Gordonia terrae* can be difficult to identify and treat. It may be especially challenging to diagnose these cases in countries with limited diagnostic resources.

**Electronic supplementary material:**

The online version of this article (doi:10.1186/s12879-017-2283-2) contains supplementary material, which is available to authorized users.

## Background

*Gordonia terrae* is a gram-positive, coccoid to bacillary, weakly acid-fast bacterium that was initially isolated from soil and is a rare cause of clinical infection. Unfortunately, the *Gordonia* spp. are not identifiable using Gram staining of clinical specimens, and definitive identification may require the use of a 16S rRNA sequence analysis. We report the first case of peritoneal dialysis-related peritonitis caused by *Gordonia terrae* in mainland China. Matrix-assisted laser desorption/ionization time-of-flight mass spectrometry initially identified the responsible strain as *Gordonia rubropertincta*, although definitive identification was achieved using a 16S rRNA sequence analysis. To the best of our knowledge, only 23 similar cases have been reported in the English literature (Table 1).

**Table 1:** Literature reports of infections caused by *Gordonia terrae* (1992–2016)

No.	Author	Pt age (years)	Pt sex	Pt Country	Clinical manifestation	Days to Dx after Ad	Dx basis	Antibiotics used prior to diagnosis	Final antibiotic regimen	Ref
1	Drancourt et al.	3	M	USA	fever, headache	24	C + SH + HPLC	Ct + M	V + To	[3]
2	Pham et al.	28	M	USA	fever, chills, headache	7–9	C + 16S	Cz	V	[4]
3	Pham et al.	44	F	USA	fever,	7–9	C + 16S	Cz	V	[4]
4	Pham et al.	54	F	USA	fever,	7–9	C + 16S	L+ Imp	V	[4]
5	Pham et al.	46	F	USA	fever, chills	7–9	C + 16S	E	V+ Imp	[4]
6	Pham et al.	60	M	USA	fever, chills, orthostatic hypotension	7–9	C + 16S	At	Cd + Az	[4]
7	Drancourt et al.	40	F	France	fever, hemiparesis	7	C + 16S	SMZ + OF	V	[12]
8	Bakker et al.	15	M	Netherlands	fever, thumb pain	14	C + 16S	D	NA	[13]
9	Gil-Sande et al.	61	M	Spain	fever, urine retention, altered mental status	20	C + 16S	PT + G	NA	[5]
10	Nicodemo et al.	55	F	Brazil	fever, nausea	15	C + 16S	PT + V	Imp + Ci	[6]
11	Lai et al.	58	F	Taiwan	fever	NA	C + 16S	Imp + Ci	V	[7]
12	Lai et al.	23	M	Taiwan	fever, bone pain	NA	C + 16S	Imp	V	[7]
13	Lai et al.	75	M	Taiwan	fever	NA	C + 16S	Ci	V	[7]
14	Lai et al.	30	M	Taiwan	fever	NA	C + 16S	AC	NA	[7]
15	Lai et al.	48	M	Taiwan	fever, cough	NA	C + 16S	AC	NA	[7]
16	Ma et al.	45	M	Hong Kong	abdominal pain	21	C + 16S	Ca + Cz	V	[10]
17	Ma et al.	61	M	Hong Kong	NA	13	C + 16S	Ci + Ca + Cz	V	[10]
18	Ma et al.	60	M	Hong Kong	NA	12	C + 16S	Ca + Cz	Mem	[10]
19	Blanc et al.	41	F	France	eye smarting	>3	C + 16S	NA	NA	[2]
20	Grisold et al.	24	M	Austria	fever, dyspnoea, nausea, and vomiting	NA	C + 16S	L + PT	NA	[14]
21	Zardawi et al.	29	F	Australia	mass in breast	>7	C + 16S	NA	Te	[15]
22	Zampella et al.	56	F	USA	mass on foot	NA	C + 16S	SMZ	AC	[16]
23	Aoyama et al.	NA	NA	Japan	pulmonary infections	NA	C + 16S	NA	NA	[17]

*Ad* admitted*, C* culture, *Dx* diagnosis, *NA* data not available, *SH* southern hybridization, *HPLC* high performance liquid chromatography, *Pt* patient, *Ref* reference, *AC* amoxicillin-clavulanate, *Az* azithromycin, *At* atreonam, *Cd* clindamycin, *Ct* ceftriaxone, *Ca* cefazolin, *Cz* ceftazidime, *Ci* ciprofloxacin, *D* doxycycline, *E* erythromycin, *G* gentamicin, *Mem* meropenem, *Imp* imipenem, *L* levofloxacin, *M* metronidazole, *OF* ofloxacin, *PT* piperacillin/tazobactam, *SMZ* sulfamethoxazole, *To* tobramycin, *Te* tetracycline, *V* vancomycin

## Case presentation

### Medical history of the patient

A 52-year-old man was diagnosed with uraemia at a local hospital in February 2013. He was treated using haemodialysis while in the hospital and with peritoneal dialysis after discharge. His serum creatinine concentration was 1000 μmol/L, showing two reduced renal volume. The patient was also diagnosed with chronic obstructive pulmonary disease, grade 3 hypertension (high-risk), and cardiac insufficiency. Starting in June 2015, the patient reported experiencing epigastric pain during the peritoneal dialysis, which was exacerbated during inspiration, relieved during dialysis fluid removal, and absent during non-dialysis periods. The dialysis fluids were smooth and accompanied by floccule.

On November 17, 2015, the patient experienced bloody ascites, swelling of both lower limbs, and asthma after playing sports and was admitted to our hospital. A physical examination revealed a body temperature of 38.0 °C, a respiratory rate of 22 breaths/min, a pulse of 110 beats/min, and blood pressure of 130/63 mmHg. The patient showed clear consciousness, but he exhibited facial signs of chronic anaemia, distension of the jugular vein, barrel chest, mild swelling of both lower limbs, wheezing in both lungs, an expanded heart boundary on two sides, abdominal wall tension, positive pressure at the navel, and no rebound tenderness. Laboratory testing revealed a white blood cell count of 3.4 × 10^9^/L, a procalcitonin level of 2.69 ng/mL, a B-type natriuretic peptide level of 460 pg/mL, a prothrombin time of 14.5 s, and a D-dimer level of 2,127 ng/mL. Routine testing of the peritoneal fluid revealed a slightly muddy appearance with a specific gravity of 1.018, weak positive results in the Rivalta test, a white blood cell count of 510 × 10^6^/L (mononuclear cells: 40%, multinuclear cells: 60%), a red blood cell count of 2,980 × 10^6^/L, and positive results for coagulability.

The patient’s peritoneal fluid was submitted for culturing, and the patient received initial treatment using intravenous cefazolin, which was changed to intraperitoneal ceftazidime and vancomycin on November 19. On November 23, the patient’s body temperature was 36.5 °C. He reported that his chest congestion and stomach ache were relieved, and the dialysis fluid appeared smooth and clear. The patient and his relatives insisted that he be discharged, despite our informing them that the culture results were incomplete, the infection might not be controlled, and the patient might experience recurrence of the peritonitis and septicopyaemia. Unfortunately, a telephone follow-up with the patient’s relatives revealed that he had not received continued treatment after his discharge and had died in February 2016. The cause of death was multiorgan failure.

### Bacteria culture and identification

**Fig. 1:**
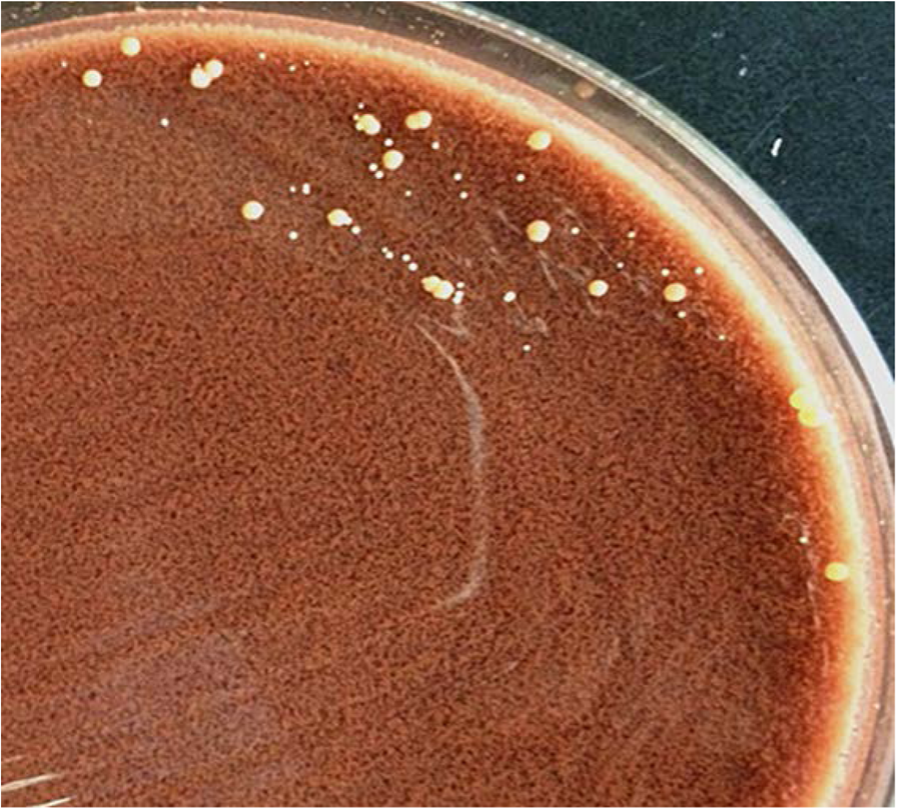
The colonies on a chocolate agar plate after 72 h of incubation

**Fig. 2:**
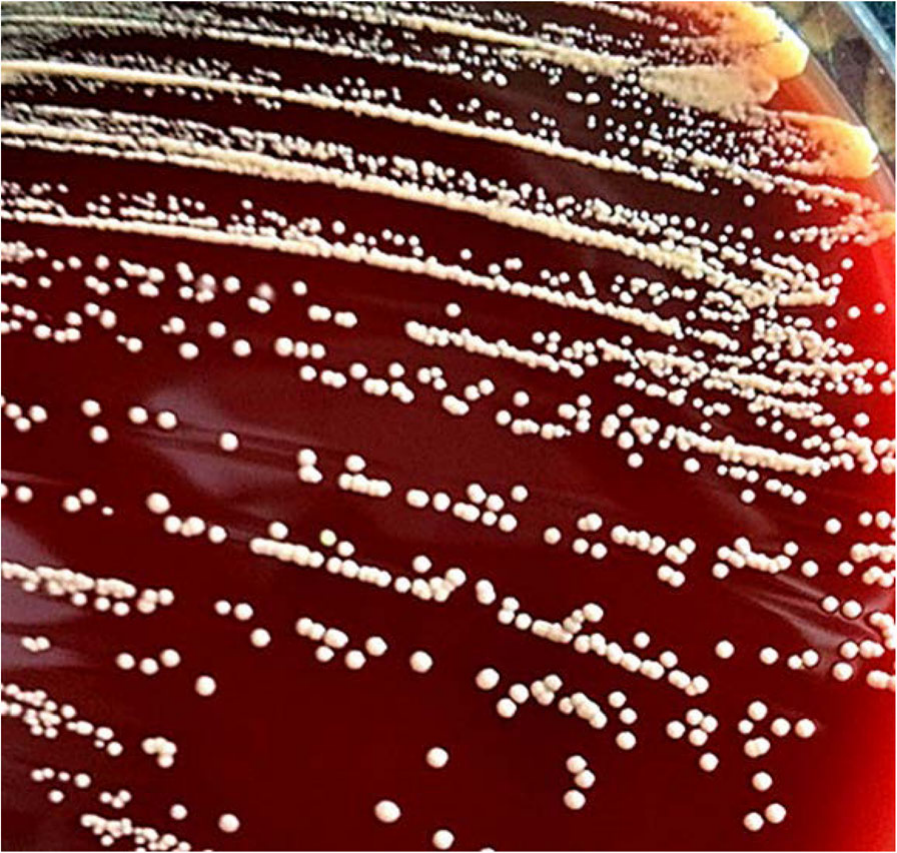
The colonies on a blood agar plate after 6 days of incubation

**Fig. 3:**
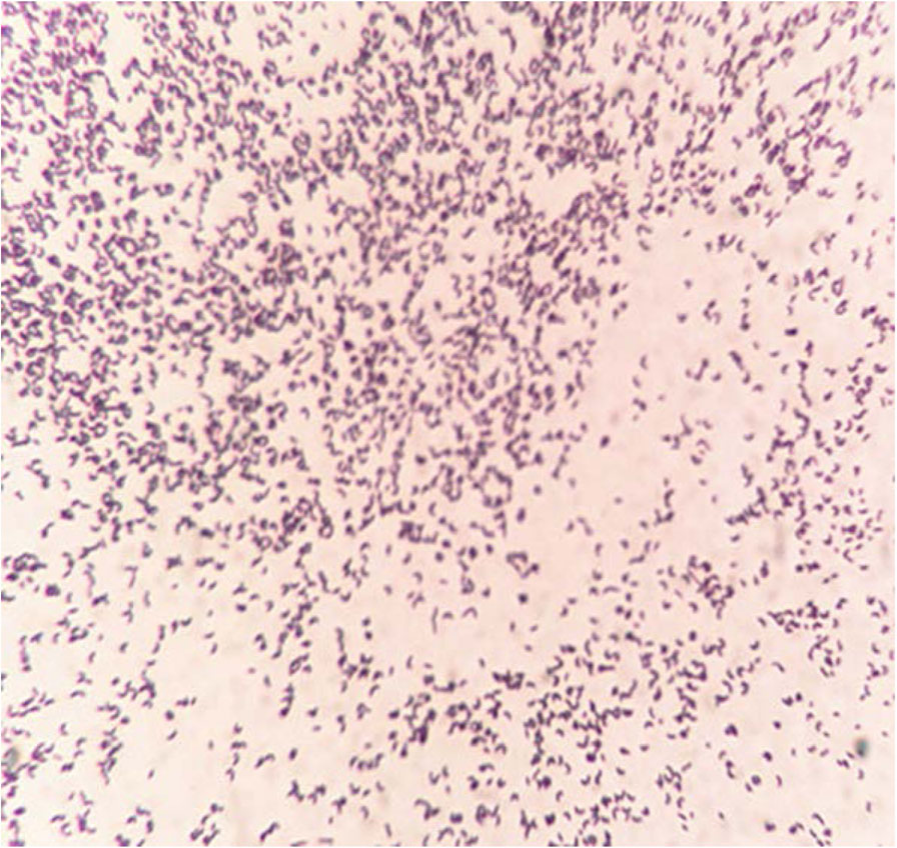
Gram staining (magnification: 1,000×)

**Fig. 4:**
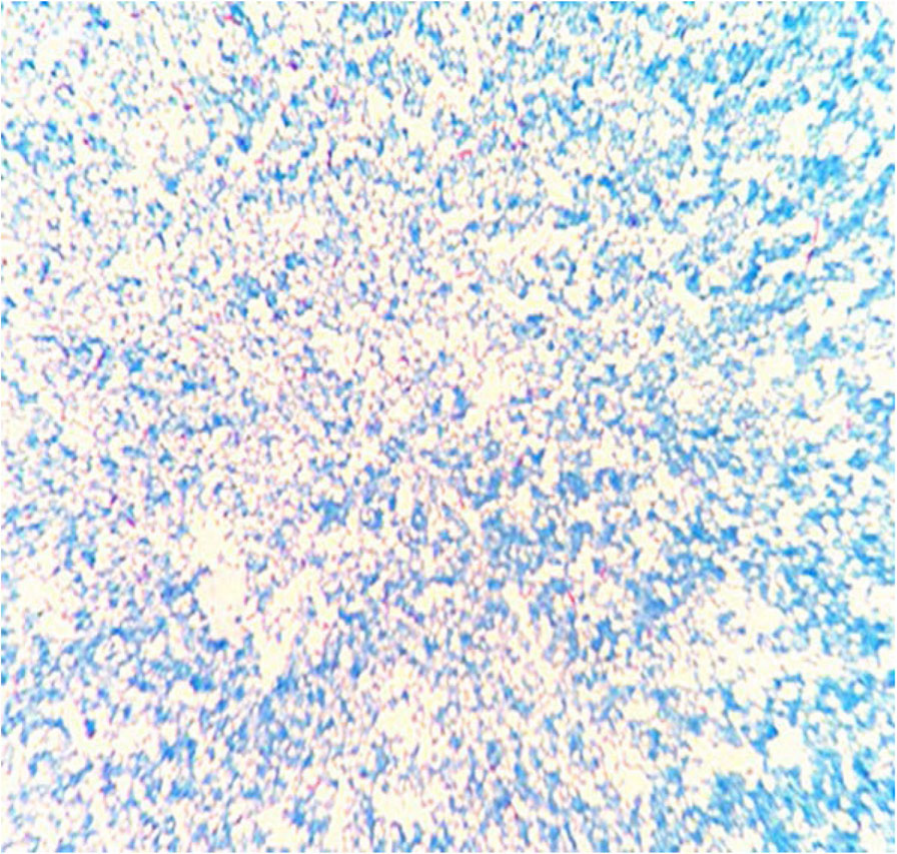
Weak acid fast staining (magnification: 1,000×)

**Fig. 5:**
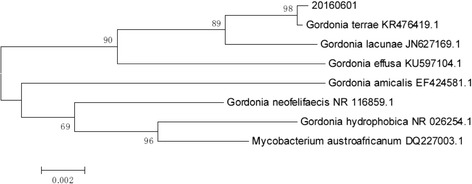
Phylogenetic tree of the isolate

On November 19, 2015, the patient’s peritoneal fluid was aseptically collected into aerobic blood culture bottles (BD, USA) and sent to a clinical laboratory, where it was cultured using the Bactec^TM^ 9120 blood culture system (BD, USA). After culturing for 51.76 h, a positive result was observed, and the culture fluid was directly smeared to identify gram-positive bacilli. The fluid was also transferred to blood agar (Zhengzhou Autobio) and chocolate agar plates (Tianjin Jinzhang), which were incubated at 35 °C with 5% CO_2_. There were no visible colonies after 24 h of culturing, although both plates exhibited small light-brown colonies after 48 h of culturing. The colonies were positive in the catalase test. After 72 h on the chocolate agar plate and 6 days on the blood agar plate, the colonies were dry, cracked, elevated, and light brown in colour (Figs. 1 and 2). Figures 3 and 4 show that the colonies were confirmed to be gram-positive bacilli with positive, weak acid-fast staining. Additional testing using the MicrolexLT/SH mass spectrometer (Bruker) identified the strain as *G. rubropertincta* with a score of 1.702. However, a 16S rRNA sequence analysis was also performed at the Shanghai Sangon Company, using the following primers: 27 F (5′-GAGTTTGATCCTGGCTCAG-3′) and 1492R (5′-AAGGAGGTGATCCAGCCGCA-3′) [1]. After Blast alignment, we observed that the bacteria were 99% similar to the sequence of *G. terrae* EY-T12 (GenBank: KR476419.1). The phylogenetic tree of the 20160601 isolate is shown in Fig. 5.

### Antimicrobial susceptibility test

An antimicrobial susceptibility testing was performed using the E-test (Zhengzhou Autobio), in which in the McFarland and M-H plates (Tianjin Jinzhang) are adjusted to 0.5 and incubated at 35 °C with 5% CO_2_ for 48 h. The results from the patient described in this report and from those in previous studies are summarized in Table 2. The strains in the previous studies were also tested using the E-test after 48 h of incubation. The isolate from the present report was resistant to ceftazidime and susceptible to penicillin, ampicillin, amikacin, erythromycin, ceftriaxone, imipenem, gentamicin, and vancomycin.

**Table 2:** Antibiotic susceptibility testing of *Gordonia terrae* in the present study and previous studies

Antibiotic	Present study (MIC, mg/mL)	Previous studies^a^ (MIC, mg/mL)
Imipenem	0.024	0.016–2.0
Gentamicin	0.38	0.125–4.0
Vancomycin	0.38	0.125–16
Ceftriaxone	0.75	0.19 to >512
Ceftazidime	>256	2.0 to >256
Penicillin	0.064	0.064 to >32
Ampicillin	0.125	0.25–32
Amikacin	0.38	0.064–0.25
Erythromycin	4	>16

^a^References [2–7, 10, 12–17]

## Discussion

The *Gordonia* genus includes 29 species, including *G. bronchialis, G. aichiensis*, *G. rubropertincta*, *G. sputi,* and *G. terrae*, which are associated with human disease [2]. *G. terrae* is an aerobic bacillus that grows slowly (a culture time of >48 h is needed) and is most frequently identified in the environment. However, *G. terrae* also infects patients with reduced immune status and causes brain abscesses, duct-associated blood infections, and cholecystitis and nephrapostasis after transplantation [3–6]. Lai et al. retrospectively identified 7 *G. terrae* strains from among 15 *Gordonia* strains from 9 cases of infection at a Taiwanese university hospital (1997–2008), and all infected patients had a reduced immune status [7].

Unfortunately, *Gordonia*, *Rhodococcus*, *Nocardia,* and *Mycobacterium* are all actinomycetes and can easily be misidentified during smear microscopic analysis and biochemical testing. In addition, *G. terrae* can metabolize rhamnose and produce acid, but cannot metabolize raffinose, which may increase the likelihood of confusing it with *Rhodococcus. Nocardia* can generate aerial hyphae on blood agar plates and provide positive results during lysozyme resistance testing, while *Mycobacterium* might also be confused with *G. terrae* based on its production of mycolic acid during high-performance liquid chromatography [8]. Therefore, the definitive identification of these bacteria should be performed using 16S rRNA sequence analysis.

Peritoneal dialysis is the main therapy for patients with end-stage renal failure, although patients with a reduced immune status may easily experience peritonitis. A 15-year retrospective analysis of infectious peritonitis associated with peritoneal dialysis in China revealed that the identification rate of gram-positive bacilli increased from 0% during 1990–2000 to 5.6% during 2001–2005 [9]. In addition, four cases of peritoneal dialysis-related peritonitis involved bacilli found to be gram-positive on the third day of culturing, with 3 cases that involved *G. terrae*. However, identification of *G. terrae* took 7–32 days, and the antibiotics were subsequently changed from cefazolin to vancomycin [10]. In the present case, the patient’s peritoneal fluid was confirmed as being gram-positive after 51.76 h of culturing, although 15 days were required to complete the definitive identification of the bacteria, which prevented us from adjusting the patient’s antibiotic treatment because he had been discharged before we obtained the definitive identification. The mass spectrometry results correctly identified the isolate as *Gordonia*, although it incorrectly identified the species. Thus, although mass spectrometry is convenient and rapid, it may not be able to accurately identify rare bacterial strains. After we confirmed the isolate’s identity using 16S rRNA sequencing, we contacted the mass spectrometry engineer and reconstructed spectrograms for the species.

Interestingly, we have not found any evidence regarding the appropriate antibiotic therapy for *Gordonia*. Johnson et al. reported that *G. terrae* is sensitive to most antibiotics (including β-lactams, glycopeptides, and carbapenems), although the treatment course is too long for patients with peritonitis [11]. For example, the previous reports regarding *G. terrae* indicated that at least 3 weeks of vancomycin therapy were needed to prevent secondary infections. Therefore, clinicians should carefully consider the development of secondary peritonitis in patients with peritoneal dialysis caused by *G. terrae*. In addition, efforts should be made to rapidly and definitively identify the responsible strain in order to facilitate effective therapy.

## Conclusions

The present case is the first report of an peritoneal dialysis-related peritonitis caused by Gordonia terrae in mainland China. The findings from our case and the previously reported cases indicate that peritoneal dialysis-related peritonitis caused by Gordonia terrae can be difficult to identify and treat. It may be especially challenging to diagnose these cases in countries with limited diagnostic resources. In addition, resistance to the antimicrobial agents were observed in isolates from the present patient as well as previous reports.

## Additional file

Additional file 1: 16S rRNA sequence analysis. (DOCX 58 kb)
