# Supplementary material for: A Chinese patient with peritoneal dialysis-related peritonitis caused by Gordonia terrae: a case report
Source: BMC Infect Dis. 2017 Feb 28;17:179. doi: 10.1186/s12879-017-2283-2 (PMC5331635; doi:10.1186/s12879-017-2283-2)
Supplement: Additional file 1: — 16S rRNA sequence analysis. (DOCX 58 kb) [file 12879_2017_2283_MOESM1_ESM.docx]

**File name:** 16S rRNA sequence analysis

**Title of data:** The result of gene identification by 16S rRNA sequence analysis

**Description of data:** a 16S rRNA sequence analysis was also performed at the Shanghai Sangon Company, using the following primers: 27F (5'-GAGTTTGATCCTGGCTCAG-3') and 1492R (5'-AAGGAGGTGATCCAGCCGCA-3') . After Blast alignment on web of, we observed that the bacteria were 99% similar to the sequence of G. terrae EY-T12 (GenBank: KR476419.1)

**S531 1343bp**

CTGGGTACTCGAGTGGCGAACGGGTGAGTAACACGTGGGTGATCTGCCCTGCACTCTGGGATAAGCCTGGGAAACTGGGTCTAATACCGGATATGACCATGGGATGCATGTTCTGTGGTGGAAAGCTTTTGCGGTGTGGGATGGGCCCGCGGCCTATCAGCTTGTTGGTGGGGTAATGGCCTACCAAGGCGACGACGGGTAGCCGACCTGAGAGGGTGATCGGCCACACTGGGACTGAGACACGGCCCAGACTCCTACGGGAGGCAGCAGTGGGGAATATTGCACAATGGGCGCAAGCCTGATGCAGCGACGCCGCGTGAGGGATGACGGCCTTCGGGTTGTAAACCTCTTTCACCAGGGACGAAGCGTGAGTGACGGTACCTGGAGAAGAAGCACCGGCCAACTACGTGCCAGCAGCCGCGGTAATACGTAGGGTGCGAGCGTTGTCCGGAATTACTGGGCGTAAAGAGCTCGTAGGCGGTTTGTCGCGTCGTCTGTGAAATTCTGCAACTCAATTGTAGGCGTGCAGGCGATACGGGCAGACTTGAGTACTACAGGGGAGACTGGAATTCCTGGTGTAGCGGTGAAATGCGCAGATATCAGGAGGAACACCGGTGGCGAAGGCGGGTCTCTGGGTAGTAACTGACGCTGAGGAGCGAAAGCGTGGGTAGCGAACAGGATTAGATACCCTGGTAGTCCACGCCGTAAACGGTGGGTACTAGGTGTGGGTTCCTTTTCACGGGATCCGTGCCGTAGCTAACGCATTAAGTACCCCGCCTGGGGAGTACGGCCGCAAGGCTAAAACTCAAAGGAATTGACGGGGGCCCGCACAAGCGGCGGAGCATGTGGATTAATTCGATGCAACGCGAAGAACCTTACCTGGGTTTGACATACACCAGACGCGGCTAGAGATAGTCGTTCCCTTGTGGTTGGTGTACAGGTGGTGCATGGCTGTCGTCAGCTCGTGTCGTGAGATGTTGGGTTAAGTCCCGCAACGAGCGCAACCCTTGTCCTGTATTGCCAGCGGGTTATGCCGGGGACTTGCAGGAGACTGCCGGGGTCAACTCGGAGGAAGGTGGGGATGACGTCAAGTCATCATGCCCCTTATGTCCAGGGCTTCACACATGCTACAATGGCTGGTACAGAGGGCTGCGATACCGTGAGGTGGAGCGAATCCCTTAAAGCCAGTCTCAGTTCGGATTGGGGTCTGCAACTCGACCCCATGAAGTCGGAGTCGCTAGTAATCGCAGATCAGCAACGCTGCGGTGAATACGTTCCCGGGCCTTGTACACACCGCCCGTCACGTCATGAAAGTCGGTAACACCCGAAGCCGGTGGCCTAAC

| **Description** | [**Max score**](http://blast.ncbi.nlm.nih.gov/Blast.cgi?CMD=Get&ALIGNMENTS=100&ALIGNMENT_VIEW=Pairwise&DATABASE_SORT=0&DESCRIPTIONS=100&DYNAMIC_FORMAT=on&FIRST_QUERY_NUM=0&FORMAT_OBJECT=Alignment&FORMAT_PAGE_TARGET=&FORMAT_TYPE=HTML&GET_SEQUENCE=yes&I_THRESH=&LINE_LENGTH=60&MASK_CHAR=2&MASK_COLOR=1&NUM_OVERVIEW=100&OLD_BLAST=false&PAGE=MegaBlast&QUERY_INDEX=0&QUERY_NUMBER=0&RESULTS_PAGE_TARGET=&RID=MRK37BB701R&SHOW_LINKOUT=yes&SHOW_OVERVIEW=yes&STEP_NUMBER=&OLD_VIEW=false&DISPLAY_SORT=1&HSP_SORT=1) | [**Total score**](http://blast.ncbi.nlm.nih.gov/Blast.cgi?CMD=Get&ALIGNMENTS=100&ALIGNMENT_VIEW=Pairwise&DATABASE_SORT=0&DESCRIPTIONS=100&DYNAMIC_FORMAT=on&FIRST_QUERY_NUM=0&FORMAT_OBJECT=Alignment&FORMAT_PAGE_TARGET=&FORMAT_TYPE=HTML&GET_SEQUENCE=yes&I_THRESH=&LINE_LENGTH=60&MASK_CHAR=2&MASK_COLOR=1&NUM_OVERVIEW=100&OLD_BLAST=false&PAGE=MegaBlast&QUERY_INDEX=0&QUERY_NUMBER=0&RESULTS_PAGE_TARGET=&RID=MRK37BB701R&SHOW_LINKOUT=yes&SHOW_OVERVIEW=yes&STEP_NUMBER=&OLD_VIEW=false&DISPLAY_SORT=2&HSP_SORT=1) | [**Query cover**](http://blast.ncbi.nlm.nih.gov/Blast.cgi?CMD=Get&ALIGNMENTS=100&ALIGNMENT_VIEW=Pairwise&DATABASE_SORT=0&DESCRIPTIONS=100&DYNAMIC_FORMAT=on&FIRST_QUERY_NUM=0&FORMAT_OBJECT=Alignment&FORMAT_PAGE_TARGET=&FORMAT_TYPE=HTML&GET_SEQUENCE=yes&I_THRESH=&LINE_LENGTH=60&MASK_CHAR=2&MASK_COLOR=1&NUM_OVERVIEW=100&OLD_BLAST=false&PAGE=MegaBlast&QUERY_INDEX=0&QUERY_NUMBER=0&RESULTS_PAGE_TARGET=&RID=MRK37BB701R&SHOW_LINKOUT=yes&SHOW_OVERVIEW=yes&STEP_NUMBER=&OLD_VIEW=false&DISPLAY_SORT=4&HSP_SORT=0) | [**E value**](http://blast.ncbi.nlm.nih.gov/Blast.cgi?CMD=Get&ALIGNMENTS=100&ALIGNMENT_VIEW=Pairwise&DATABASE_SORT=0&DESCRIPTIONS=100&DYNAMIC_FORMAT=on&FIRST_QUERY_NUM=0&FORMAT_OBJECT=Alignment&FORMAT_PAGE_TARGET=&FORMAT_TYPE=HTML&GET_SEQUENCE=yes&I_THRESH=&LINE_LENGTH=60&MASK_CHAR=2&MASK_COLOR=1&NUM_OVERVIEW=100&OLD_BLAST=false&PAGE=MegaBlast&QUERY_INDEX=0&QUERY_NUMBER=0&RESULTS_PAGE_TARGET=&RID=MRK37BB701R&SHOW_LINKOUT=yes&SHOW_OVERVIEW=yes&STEP_NUMBER=&OLD_VIEW=false&DISPLAY_SORT=0&HSP_SORT=0) | [**Ident**](http://blast.ncbi.nlm.nih.gov/Blast.cgi?CMD=Get&ALIGNMENTS=100&ALIGNMENT_VIEW=Pairwise&DATABASE_SORT=0&DESCRIPTIONS=100&DYNAMIC_FORMAT=on&FIRST_QUERY_NUM=0&FORMAT_OBJECT=Alignment&FORMAT_PAGE_TARGET=&FORMAT_TYPE=HTML&GET_SEQUENCE=yes&I_THRESH=&LINE_LENGTH=60&MASK_CHAR=2&MASK_COLOR=1&NUM_OVERVIEW=100&OLD_BLAST=false&PAGE=MegaBlast&QUERY_INDEX=0&QUERY_NUMBER=0&RESULTS_PAGE_TARGET=&RID=MRK37BB701R&SHOW_LINKOUT=yes&SHOW_OVERVIEW=yes&STEP_NUMBER=&DISPLAY_SORT=3&HSP_SORT=3) | **Accession** |
| --- | --- | --- | --- | --- | --- | --- |
| [Gordonia sp. MDT1-31-2 16S ribosomal RNA gene, partial sequence](http://blast.ncbi.nlm.nih.gov/Blast.cgi#alnHdr_422740613) | 2481 | 2481 | 100% | 0.0 | 100% | [JX949623.1](http://www.ncbi.nlm.nih.gov/nucleotide/422740613?report=genbank&log$=nucltop&blast_rank=1&RID=MRK37BB701R) |
| [Gordonia terrae strain EY-T12 16S ribosomal RNA gene, partial sequence](http://blast.ncbi.nlm.nih.gov/Blast.cgi#alnHdr_937501600) | 2475 | 2475 | 100% | 0.0 | 99% | [KR476419.1](http://www.ncbi.nlm.nih.gov/nucleotide/937501600?report=genbank&log$=nucltop&blast_rank=2&RID=MRK37BB701R) |
| [Gordonia terrae strain EY-T8 16S ribosomal RNA gene, partial sequence](http://blast.ncbi.nlm.nih.gov/Blast.cgi#alnHdr_937501596) | 2475 | 2475 | 100% | 0.0 | 99% | [KR476415.1](http://www.ncbi.nlm.nih.gov/nucleotide/937501596?report=genbank&log$=nucltop&blast_rank=3&RID=MRK37BB701R) |
| [Gordonia terrae strain P108 16S ribosomal RNA gene, partial sequence](http://blast.ncbi.nlm.nih.gov/Blast.cgi#alnHdr_1012904530) | 2464 | 2464 | 100% | 0.0 | 99% | [KU597152.1](http://www.ncbi.nlm.nih.gov/nucleotide/1012904530?report=genbank&log$=nucltop&blast_rank=4&RID=MRK37BB701R) |
| [Gordonia terrae strain Siri-Phe4 16S ribosomal RNA gene, partial sequence](http://blast.ncbi.nlm.nih.gov/Blast.cgi#alnHdr_408360416) | 2464 | 2464 | 100% | 0.0 | 99% | [JX500280.1](http://www.ncbi.nlm.nih.gov/nucleotide/408360416?report=genbank&log$=nucltop&blast_rank=5&RID=MRK37BB701R) |
| [Gordonia terrae strain C16-Siri101 16S ribosomal RNA gene, partial sequence](http://blast.ncbi.nlm.nih.gov/Blast.cgi#alnHdr_408360402) | 2464 | 2464 | 100% | 0.0 | 99% | [JX500266.1](http://www.ncbi.nlm.nih.gov/nucleotide/408360402?report=genbank&log$=nucltop&blast_rank=6&RID=MRK37BB701R) |
| [Gordonia terrae strain NR_61 16S ribosomal RNA gene, partial sequence](http://blast.ncbi.nlm.nih.gov/Blast.cgi#alnHdr_697995360) | 2431 | 2431 | 100% | 0.0 | 99% | [KM113032.1](http://www.ncbi.nlm.nih.gov/nucleotide/697995360?report=genbank&log$=nucltop&blast_rank=7&RID=MRK37BB701R) |
| [Gordonia sp. Sn-18 16S ribosomal RNA gene, partial sequence](http://blast.ncbi.nlm.nih.gov/Blast.cgi#alnHdr_666335891) | 2431 | 2431 | 100% | 0.0 | 99% | [KJ742899.1](http://www.ncbi.nlm.nih.gov/nucleotide/666335891?report=genbank&log$=nucltop&blast_rank=8&RID=MRK37BB701R) |
